# Superconductivity in HfTe_5_ across weak to strong topological insulator transition induced via pressures

**DOI:** 10.1038/srep44367

**Published:** 2017-03-16

**Authors:** Y. Liu, Y. J. Long, L. X. Zhao, S. M. Nie, S. J. Zhang, Y. X. Weng, M. L. Jin, W. M. Li, Q. Q. Liu, Y. W. Long, R. C. Yu, C. Z. Gu, F. Sun, W. G. Yang, H. K. Mao, X. L. Feng, Q. Li, W. T. Zheng, H. M. Weng, X. Dai, Z. Fang, G. F. Chen, C. Q. Jin

**Affiliations:** 1 Institute of Physics & School of Physics of University of Chinese Academy of Sciences, Chinese Academy of Sciences, Beijing 100190, China; 2 Center for High Pressure Science & Technology Advanced Research, Shanghai, 201203, China; 3 Department of Materials Science, Jilin University, Changchun 130012, China; 4 Collaborative Innovation Center of Quantum Matter, Beijing, China

## Abstract

Recently, theoretical studies show that layered HfTe_5_ is at the boundary of weak & strong topological insulator (TI) and might crossover to a Dirac semimetal state by changing lattice parameters. The topological properties of 3D stacked HfTe_5_ are expected hence to be sensitive to pressures tuning. Here, we report pressure induced phase evolution in both electronic & crystal structures for HfTe_5_ with a culmination of pressure induced superconductivity. Our experiments indicated that the temperature for anomaly resistance peak (Tp) due to Lifshitz transition decreases first before climbs up to a maximum with pressure while the Tp minimum corresponds to the transition from a weak TI to strong TI. The HfTe_5_ crystal becomes superconductive above ~5.5 GPa where the Tp reaches maximum. The highest superconducting transition temperature (Tc) around 5 K was achieved at 20 GPa. Crystal structure studies indicate that HfTe_5_ transforms from a Cmcm phase across a monoclinic C2/m phase then to a P-1 phase with increasing pressure. Based on transport, structure studies a comprehensive phase diagram of HfTe_5_ is constructed as function of pressure. The work provides valuable experimental insights into the evolution on how to proceed from a weak TI precursor across a strong TI to superconductors.

The topological quantum materials, such as topological insulators (TIs), Dirac and Weyl semimetals, attracted much interests recently due to their rich physics and promising prospects for application in electronic and spintronic devices^1^,^2^,^3^,^4^,^5^,^6^,^7^,^8^,^9^,^10^,^11^,^12^,^13^,^14^,^15^,^16^,^17^,^18^. There is an ongoing effort to search for new materials which might host similar electronic and topological properties. Graphene, a single sheet of carbon atoms, which hosts two-dimensional (2D) Dirac fermions, was firstly proposed to be one of the quantum spin Hall (QSH) insulators^3^, but it is not suitable for application due to its small energy gap. The transition-metal pentatellurides, HfTe_5_ or ZrTe_5_, long been known as thermoelectric materials, have stimulated considerable interest and active studies due to their unusual transport properties, in which the resistivity exhibits a pronounced peak at ~80 K for HfTe_5_ and ~130 K for ZrTe_5_, respectively^19^,^20^,^21^,^22^. Recent theoretical calculations predicted that the single-layer HfTe_5_ and ZrTe_5_ are QSH insulators with large energy gap. Remarkably, the topological band structures of their 3D stacked compounds could be tuned by lattice parameters^23^. Besides, the combined scanning tunneling microscopy/spectroscopy (STM/S) and angle-resolved photoemission spectroscopy (ARPES) results demonstrate that the top monolayer of ZrTe_5_ crystals is a large-gap 2D TI^24^. This predication renewed the interests in exploring exotic quantum physical phenomena in experiments. Very recently, Li. *et al*. reported the observation of chiral anomaly in the bulk single crystal ZrTe_5_ through magneto-transport study^25^, which is similar to that observed in 3D Dirac semimetal Na_3_Bi^18^. With applying pressure, superconductivity with Tc ~ 2.5 K appears in ZrTe_5_ above 6 GPa^26^. The theoretical calculations predicate that both ZrTe_5_ and HfTe_5_ are located in the vicinity of a transition between weak and strong TI^23^. ARPES results of bulk ZrTe_5_ also suggest the possible transition from weak TI(WTI) to strong TI(STI) via pressure^27^. However, there are few research works on HfTe_5_ due to the difficulty of growing large and high quality single crystals^28^. Our preliminary study on HfTe_5_ shows very different quantum physical behaviors at ambient pressure in spite of the two compounds possess the same crystal structure^28^. These interesting results make HfTe_5_ a potential material for the study of the novel topological quantum phenomenon and topological phase transitions as function of pressures.

High pressure is a neat but powerful method^29^,^30^,^31^,^32^,^33^,^34^,^35^ to tune the electronic and crystal structures of emergent quantum matters with advantages of without introducing disorder or impurity that are always inherent to chemical doping. In this work, we report the discovery of pressure induced superconductivity in HfTe_5_ single crystals. Transport experiments indicate consecutive transitions induced by pressure from semiconductor to metal before superconductivity appears at a critical pressure of ~5.5 GPa. A systematic phase diagram on crystal and electronic properties of HfTe_5_ as a function of pressure is constructed.

## Results and Discussion

**Figure 1:**
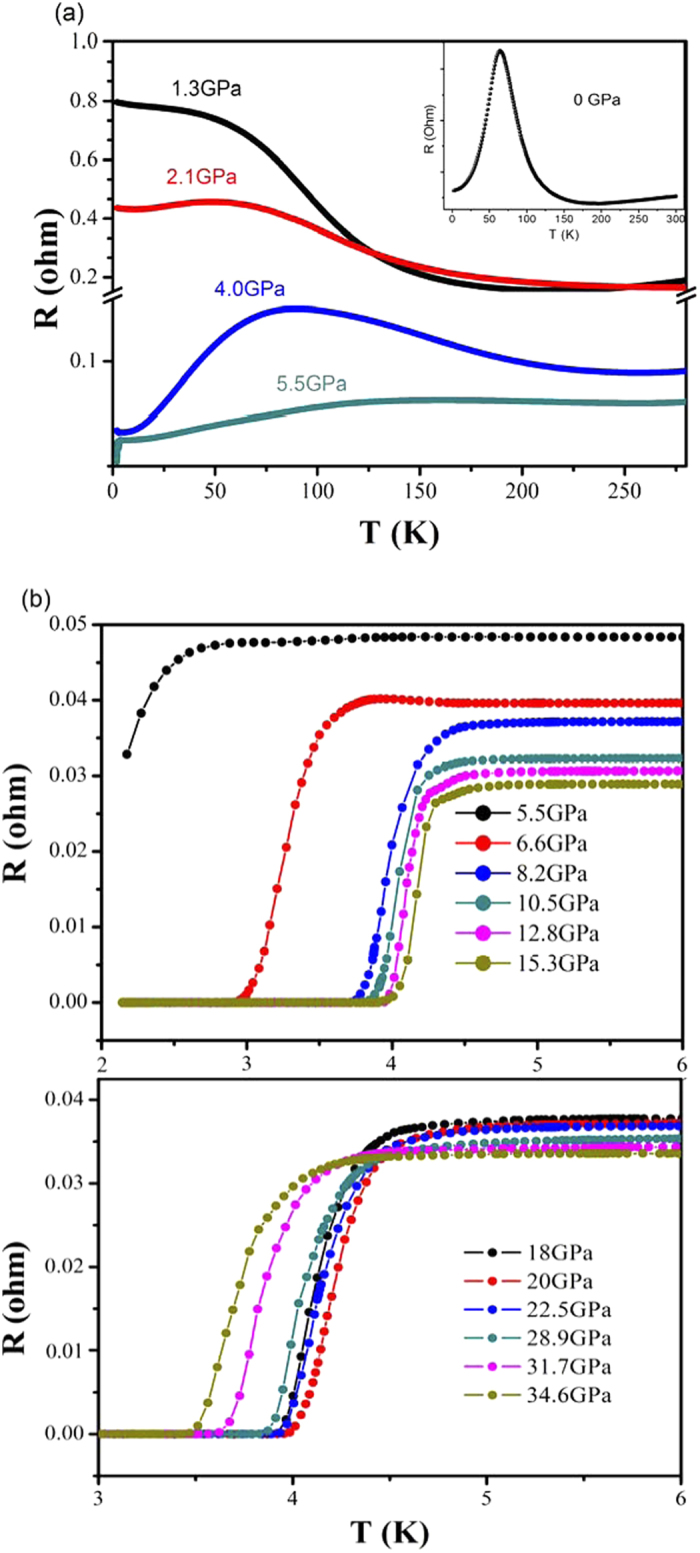
Electrical transport properties of HfTe_5_ single crystal. (**a**) Temperature dependence of *ac* plane resistance at low pressure. (**b**) The *ac* plane resistance as a function of temperature at various pressures showing superconducting transitions at high pressure.

Figure 1 shows the evolution of *ac* plane resistance as a function of temperature of HfTe_5_ single crystals at various pressures. At 1.3 GPa, the resistance displays a typical semiconductor like behavior above 40 K. As temperature continues to decrease, the resistance increases much slowly. When pressure increases up to 2.1 GPa, the resistance shows a hump near 49 K, and then decreases with temperature, accompanied by an upturn below 11 K. The behaviors of the abnormal resistance appearing at 40 K and 49 K are intimately tied to the band structure evolution with temperatures, which are similar to those observed at ambient pressure^19^,^20^,^28^. The temperature with peak resistance (Tp) increases to 84 K at 4.0 GPa, accompanied by the broadening of the hump and the decrease of the peak resistance. Up to 5.5 GPa, in addition to the increases of Tp to 136 K, a small drop of resistance is observed at low temperature which signifies the occurrence of superconducting.

Both HfTe_5_ and ZrTe_5_ display a resistive abnormal hump. The Tp in HfTe_5_ crystal decreases with pressure up to 1.7 GPa but those of ZrTe_5_ is on the opposite^36^. This results in the reduced Tp of HfTe_5_ under low pressure than that at ambient pressure (65 K) as seen in the insert of Fig. 1(a). Our experiment indicates that Tp changed systematically with pressure, showing the anomaly resistance peak moves to low temperature first before reverses to high temperature then followed by disappearance. That is in opposite to the effect of pressure on ZrTe_5_^26^.

**Figure 2:**
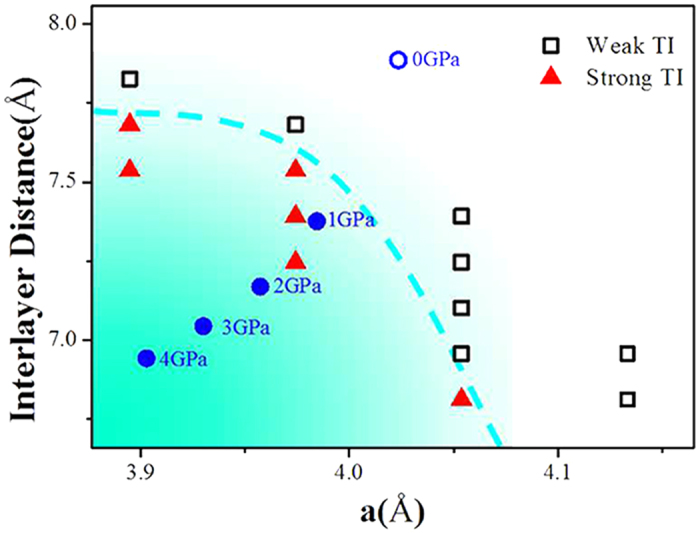
The calculated transition between weak TI and strong TI via lattice parameters change and pressure. The black empty square represents weak TI state and red solid triangle represents strong TI state based. The blue circle stands for lattice parameters at different pressure.

Due to weak interlayer coupling strength, both ZrTe_5_ and HfTe_5_ locate at the vicinity between weak and strong TI^23^, as confirmed by ARPES experiments on bulk ZrTe_5_^27^. The identification of a temperature induced Lifshitz transition directly accounts to the origin of the transport property anomalies in ZrTe_5_^27^. ARPES revealed two branches of bands near the Г point of ZrTe_5_: the upper branch (UB) above the Fermi level corresponds to electron like conduction band, and the lower branch (LB) band corresponds to the hole like valence band. There is a clear Lifshitz transition that occurs across 135 K where the Fermi surface topology transforms from an electron like pocket at low temperature to a hole like pocket at high temperature. This Lifshitz transition corresponds to the band structure where the energy gap center just crosses the Fermi level^27^. Assuming the same scenario to HfTe_5_, while the bands shift with increasing temperature, high pressure will reduce its energy gap, resulting into lower temperature where Fermi level crosses the gap center. In other word, the temperature of the resistance hump decreases with pressure first. With further increasing pressure, the enhanced interlayer coupling will transform the state from a weak TI to a strong TI thus Tp increases via the pressure. In Weng’s work^23^, they show that the stacked 3D ZrTe_5_ compound is located at the vicinity of a transition between strong and weak TI. Only the 2% change of lattice parameter will cause this transition. This can be realized through compression for HfTe_5_ as shown in Fig. 2. With the pressure increased, the topological state gradually crossed the boundary of weak and strong TI. This is in consistent with the anomaly shift of Tp via pressure shown in Fig. 1.

Further increasing pressure, the maximum of resistance is totally suppressed and the overall resistance shows a metallic transport behavior. A superconducting transition with signature of resistance drop at around 2.7 K was observed at 5.5 GPa, as shown in Fig. 1(b). The transition temperatures (Tc) was defined based on the differential of resistance over temperature (dR/dT)^29^. With pressure increasing to 6.6 GPa, Tc grows rapidly with resistance drop getting more pronounced and the zero resistance starting to be fully realized. The superconductivity transitions at pressures up to 35 GPa are shown in Fig. 1(b). In the whole pressure range, the highest Tc is achieved at about 5 K, while Tc descends slightly above 20 GPa.

**Figure 3:**
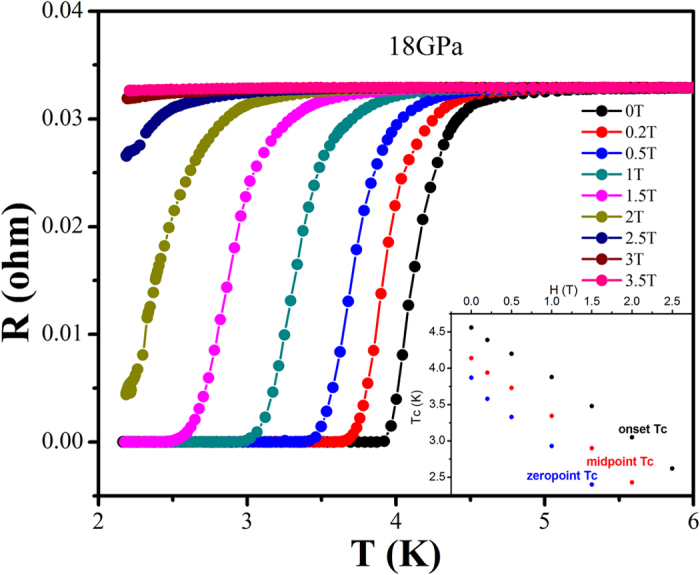
The superconducting transitions of HfTe_5_ with applied magnetic field H perpendicular to the *ac* plane of HfTe_5_ single crystal at 18 GPa. The inset shows Tc evolution as function of magnetic field H.

To assure the drop observed in Fig. 1(b) is indeed a superconducting transition, we further measured the resistance versus temperature at variant applied magnetic field(H). The evolutions of Tc at 18 GPa as a function of magnetic field are performed, as shown in Fig. 3, with insets showing the change of Tc with H. It is obvious that Tc shifts toward lower temperature with magnetic field, indicating the transition is superconductivity in nature. According to the Werthamer-Helfand-Hohenberg (WHH) formula^30^, *H*_*C2*_(*0) *=* −0.691[dH*_*C2*_(*T)/dT]*_T=Tc_*T_*C*_, the upper critical field *H*_*C*_*2(0*) is extrapolated to be 4.1 T with Tc onset, 3.4 T with Tc midpoint and 2.8 T with zero point of Tc at 18 GPa with magnetic field H paralleling to *b* axis of HfTe_5_ single crystal.

**Figure 4:**
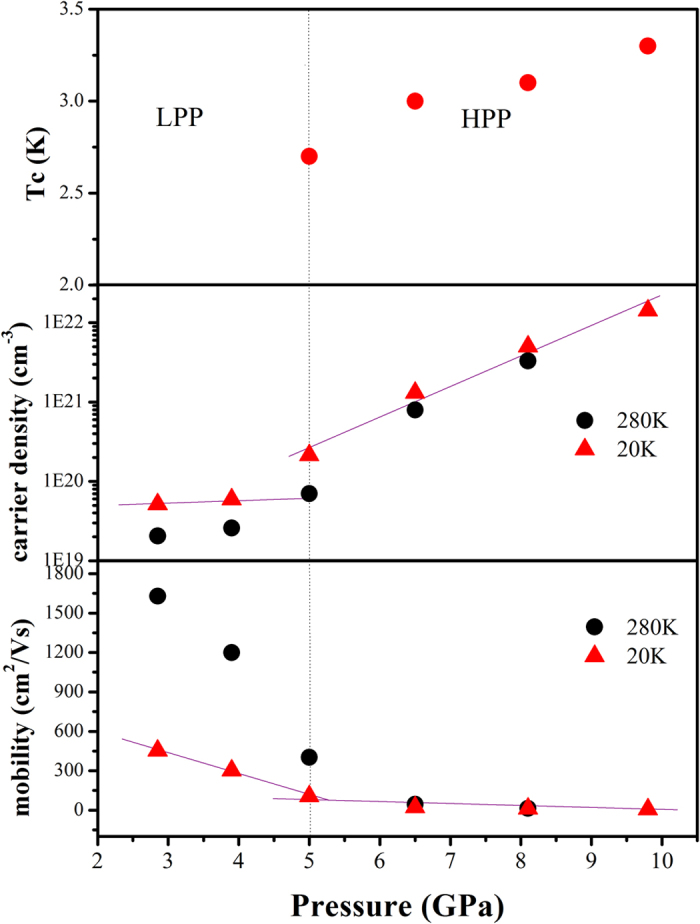
Pressure tuned changes on Tc, carrier density and mobility in HfTe_5_ at various temperatures (LPP & HPP indicate low pressure phase & high pressure phase, respectively).

To determine the carrier density we conducted Hall Effect measurements with a magnetic field H perpendicular to *ac* plane of HfTe_5_ single crystal using Van der Pauw method. Carrier density increases almost three orders of magnitude with pressure up to 9.8 GPa, as shown in Fig. 4. It is visual that carrier density increases much faster above 5 GPa than that at lower pressure, which coincides with occurrence of superconductivity. In other world, the variations of Tc with pressure are closely related to the change of carrier density or mobility. The carrier is found to be n-type like in the whole range of pressure which might be the results of two carriers competing.

**Figure 5:**
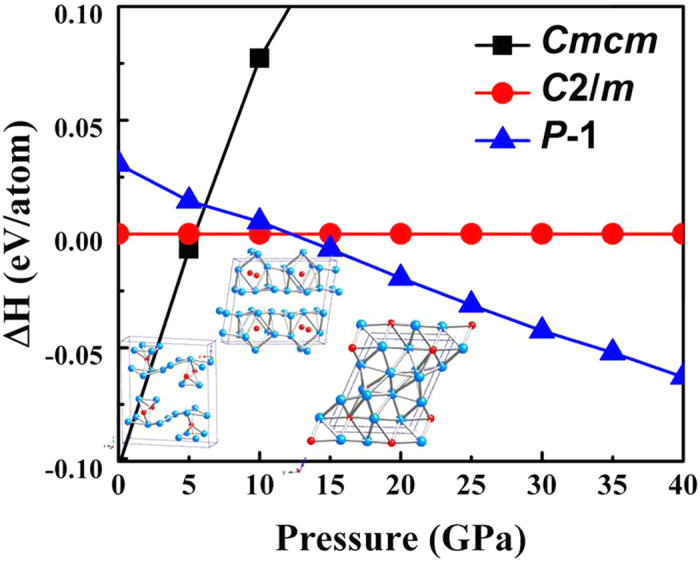
Calculated enthalpies per atom as functions of pressure up to 40 GPa.

We performed crystal structure studies based on first-principle calculations on HfTe_5_ at pressure up to 40 GPa. The enthalpies of the newly predicted stable phases, calculated at the high level of accuracy, are plotted as a function of pressure as shown in Fig. 5. The ambient pressure *Cmcm* structure is the most stable phase up to 5 GPa, followed by a phase transition to a monoclinic *C2/m* structure, which corresponds to the appearance of the superconductivity at 5.5 GPa in the transport measurements. Beyond 12 GPa, triclinic *P-1* structure becomes the most stable phase at least up to 40 GPa. The crystal structures of *C2/m* and *P-1* are shown in the inset of Fig. 5, respectively. In considerations of transport experiments, the occurrence of superconductivity is possibly related to the transition from *Cmcm* to monoclinic *C2/m*. The orthorhombic *Cmcm* phase is a layered structure with the interlayer distance of 6.9 Å at 5 GPa. Upon compression, the new phase of monoclinic *C2/m* phase is also of layered structure but with reduced interlayer distance to 3.4 Å at 6 GPa. The interlayer distance along the stacking direction decreases due to the volume shrink. The second high pressure phase with triclinic *P-1* symmetry is a compacted cubic like structure.

To further study the structure stability and the predicted new phases, we conducted *in situ* high-pressure synchrotron X-ray diffraction measurement on the HfTe_5_ powder sample as shown in Figure S3. New peaks marked with star appeared at 4.69 GPa that indicated a phase transition in well consistent with the theoretical calculations. At higher pressure region the reduced pattern intensity & resolution don’t allow track on the further phase transition.

We further studied the electronic structure of HfTe_5_ via first-principle calculations by taking into account spin orbital coupling (SOC). Figure S2 shows that HfTe_5_ is a weak topological insulator at ambient, but transforms to a metal with complicated Fermi surface at high pressures as revealed by the electronic structures at 10 GPa and 20 GPa, respectively.

**Figure 6:**
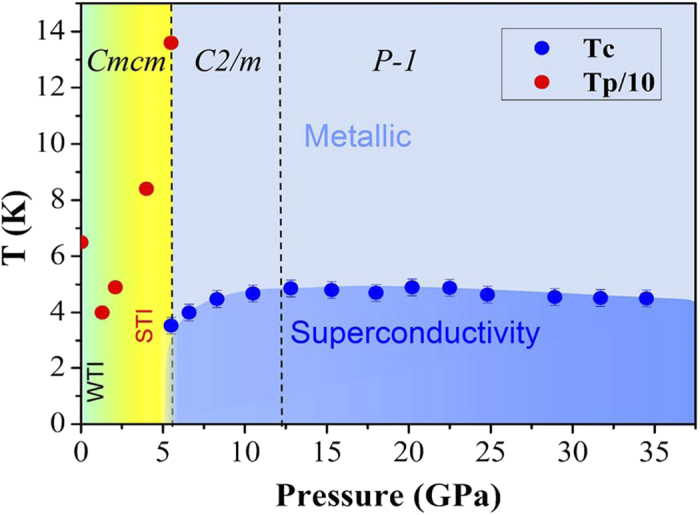
The phase diagram of HfTe_5_ single crystal as function of pressure. Tp denotes the peak temperature of resistance anomaly. The red circle represents Tp/10. The blue circle stands for the onset temperature of resistance drop. The yellow region corresponds to TI phase till 5.5 GPa. Above 5.5 GPa, the blue area indicates superconducting phase.

Referring to the results of electrical transport and predicted structure at high pressures, the phase diagram of HfTe_5_ as function of pressures is built as shown in Fig. 6. HfTe_5_ remains the ambient structure below 5.5 GPa with *Cmcm* symmetry but changes from weak topological character to strong topological character at around 1 GPa. The abnormal peak temperature Tp of resistance forms a minimum valley due to the weak TI to strong TI transition. The Tp reaches highest value ~136 K at 5.5 GPa, while superconductivity occurs. The superconductivity is stable in the pressure range at least up to 35 GPa, with the highest Tc ~ 5 K at 20 GPa.

## Conclusion

In summary superconductivity is discovered following the pressure driven transition from a weak Tl to a strong Tl in HfTe_5_ single crystal.

## Methods

### Sample synthesis and characterization

Single crystals of HfTe_5_ were grown by chemical vapor transport. Stoichiometric amounts of Hf (powder, 3 N, Zr nominal 3%) and Te (powder, 5 N) were sealed in a quartz ampoule with iodine (7 mg/mL). Quartz ampoule was placed in a two-zone furnace for almost one month with typical temperature gradient from 500 °C to 400 °C applied. HfTe_5_ single crystals present long ribbon shape^28^. The crystal structure of HfTe_5_ has been determined by powder X-ray diffraction experiments^22^, which is orthorhombic with space group of *Cmcm* as shown in Figure S1. Trigonal prismatic chains of HfTe_3_ run along a axis, and these prismatic chains are linked via parallel zigzag chains of Te atoms along the *c* axis to form a 2D sheet of HfTe_5_ in the *ac* plane. The sheets of HfTe_5_ stack along the *b* axis, forming a layered structure^23^.

### High-pressure transport measurements

The transport properties of HfTe_5_ single crystals at high pressure are measured using the standard four-probe method by diamond anvil cell (DAC) made of nonmagnetic BeCu alloy as described in refs 29 and 31, 32, 33, 34, 35. Pressure was generated by a pair of diamonds with 500 μm culet. A T301 stainless steel gasket, pre-indented from 250 μm to 30 μm thickness, was drilled a center hole with 250 μm in diameter. The gasket was then covered by cubic BN insulator layer to protect electrode from short circuit to gasket. A center hole with a diameter of 100 μm was further drilled at the insulating layer to serve as sample chamber. The HfTe_5_ single crystal with a dimension of 80 μm * 80 μm * 10 μm was loaded into sample chamber with soft NaCl fine powder as pressure transmitting medium. Slim gold wires of 18 mm in diameter are used as electrodes. Pressure was calibrated by ruby fluorescence shift method for all the experiments. The DAC was placed inside a MagLab system to perform the electric transport experiments^35^. To ensure equilibrium, the temperature was automatically controlled by the MagLab system with slow temperature change rate. A thermometer located around the sample in the diamond anvil cell was used to monitor sample temperature.

### High-pressure synchrotron XRD experiments

The high pressure X-ray diffraction experiments are conducted with a symmetric DAC. The similar procedures to transport measurements are adopted. The X-ray diffraction experiments at high pressure with synchrotron source are performed at HPCAT of Advanced Photon Source in Argonne National Laboratory with a wavelength of 0.4246 Å using a symmetric Mao Bell diamond anvil cell at room temperature. The XRD patterns are collected with a MAR 3450 image plate detector and integrated from the images by using the FIT2d software.

### High-pressure structure evolution and electronic band calculation

The structure search simulations are performed through the CALYPSO method, which is specially designed for global structural minimization unbiased by any known structural information. The first principles calculations have been carried out by using the projector augmented wave (PAW) method implemented in Vienna ab initio simulation package (VASP). The lattice parameters determined by X-ray diffraction are adopted in our calculations. Generalized gradient approximation (GGA) of Perdew-Burke-Ernzerhof type is used. The k-point sampling grids are set to 14 * 14 * 8, 11 * 11 * 7 and 11 * 7 * 3 for the self-consistent calculations of HfTe_5_ in 0 GPa, 10 GPa and 20 GPa, respectively. The cut-off energy for the plane wave expansion is chosen as 500 eV. Spin-orbit coupling (SOC) is taken into account self-consistently.

Note added: During the submission, we became aware the work reported by Y. Qi *et al*.^34^. Both works are uploaded to arXiv within three days (arXiv: 1602.08616 & arXiv: 1603.00514).

## Additional Information

**How to cite this article:** Liu, Y. *et al*. Superconductivity in Hf Te_5_ across weak to strong topological insulator transition induced via pressures. *Sci. Rep.* **7**, 44367; doi: 10.1038/srep44367 (2017).

**Publisher's note:** Springer Nature remains neutral with regard to jurisdictional claims in published maps and institutional affiliations.

## Supplementary Material

Supplementary Information
