# Supplementary material for: Superconductivity in HfTe5 across weak to strong topological insulator transition induced via pressures
Source: Sci Rep. 2017 Mar 16;7:44367. doi: 10.1038/srep44367 (PMC5353664; doi:10.1038/srep44367)
Supplement: Supplementary Information [file srep44367-s1.doc]

***Supplemental Materials***

**Superconductivity in HfTe_5_ across weak to strong topological insulator transition induced via pressures**

Y. Liu^1#^, Y.J. Long^1#^, L.X. Zhao^1^, S.M. Nie^1^, S.J. Zhang^1^, Y.X. Weng^1^, M.L. Jin^1^, W.M. Li^1^, Q.Q. Liu^1^, Y.W. Long^1^, R.C.Yu^1^, C.Z. Gu^1^, F. Sun^1^, W.G.Yang^4^, H.K. Mao^4^, X.L. Feng^3^, Q Li^3^, W.T. Zheng^3^, H.M. Weng^1.2^, X. Dai^1,2^, Z. Fang^1,2^, G.F. Chen^1,2*^, C.Q. Jin^1,2*^

1. Institute of Physics, Chinese Academy of Sciences, Beijing 100190, China

2. Collaborative Innovation Center of Quantum Matter, Beijing, China

3. Department of Materials Science, Jilin University, Changchun 130012

4. Center for High Pressure Science & Technology Advanced Research, Shanghai, 201203, China,

^^ These authors contribute equally to this work.

Correspondence and requests for materials should be addressed to C. Q. Jin (email: [Jin@iphy.ac.cn](mailto:Jin@iphy.ac.cn)) or G. F. Chen. (email: gfchen@iphy.ac.cn).


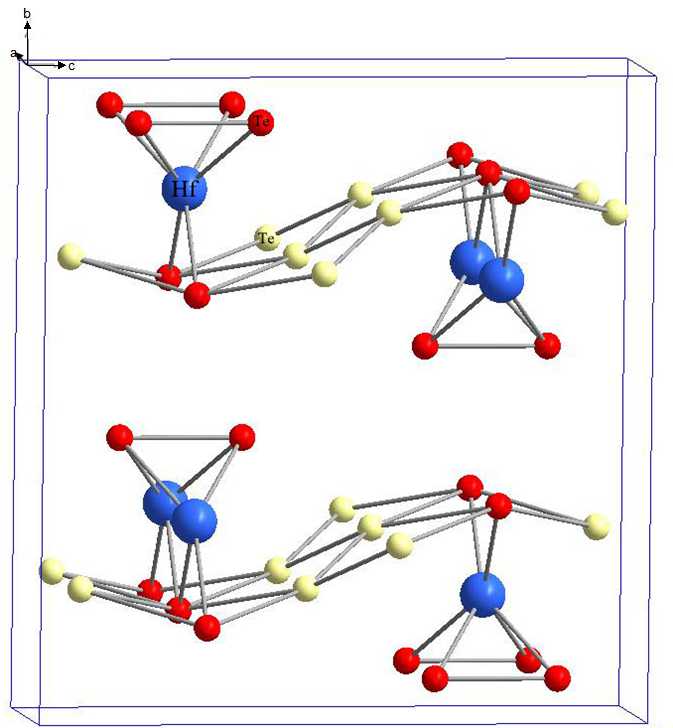


**Figure S1**: Schematic drawing of the crystallography of HfTe_5_, where blue balls stand for Hf, red and yellow balls for different type of Te.

**
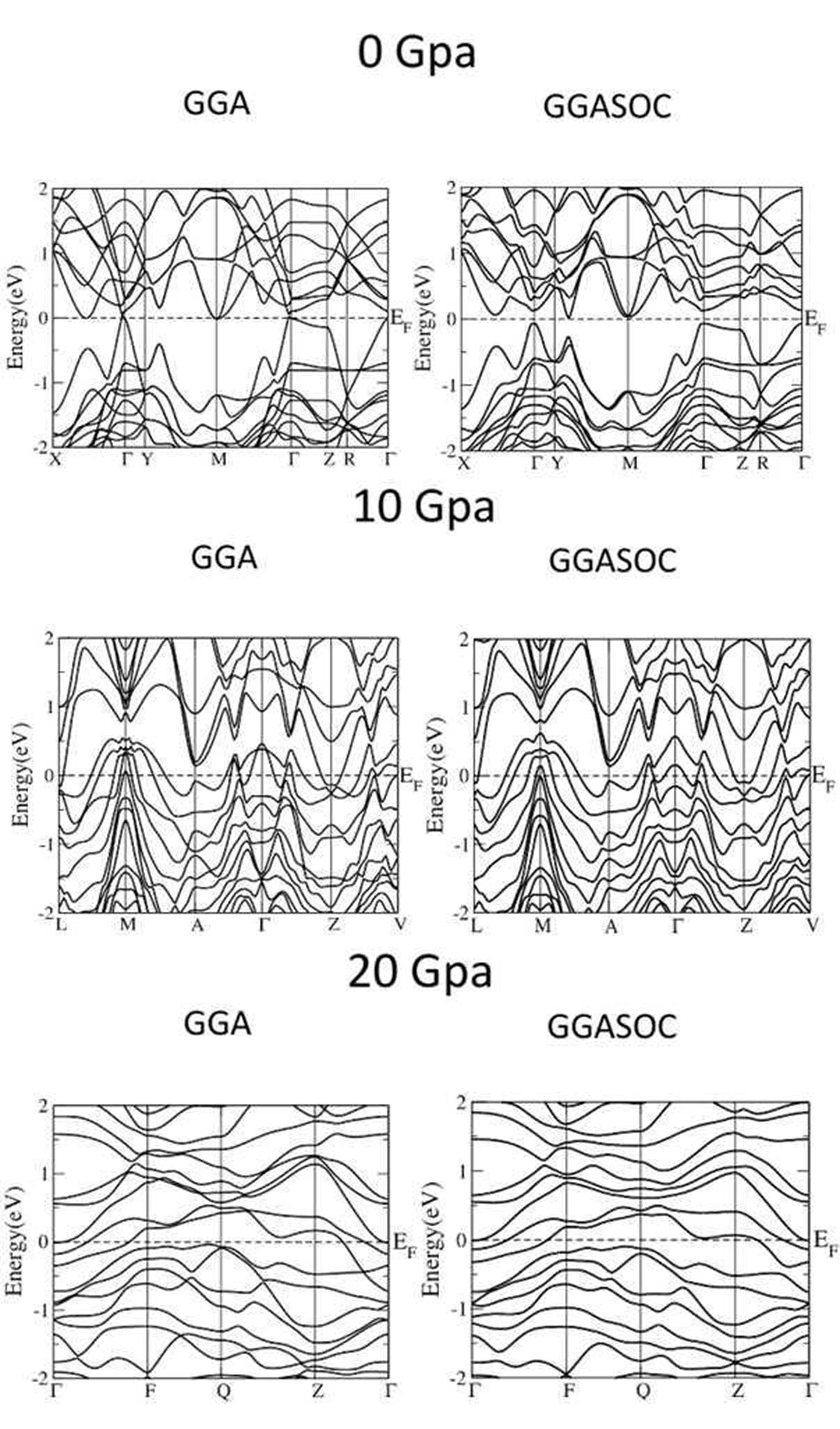
**

**Figure S2:** The calculated band structure of HfTe_5_ without and with SOC indicating a weak topological insulator at ambient, but transforms to a metal with complicated Fermi surface at high pressures at 10GPa and 20GPa, respectively.


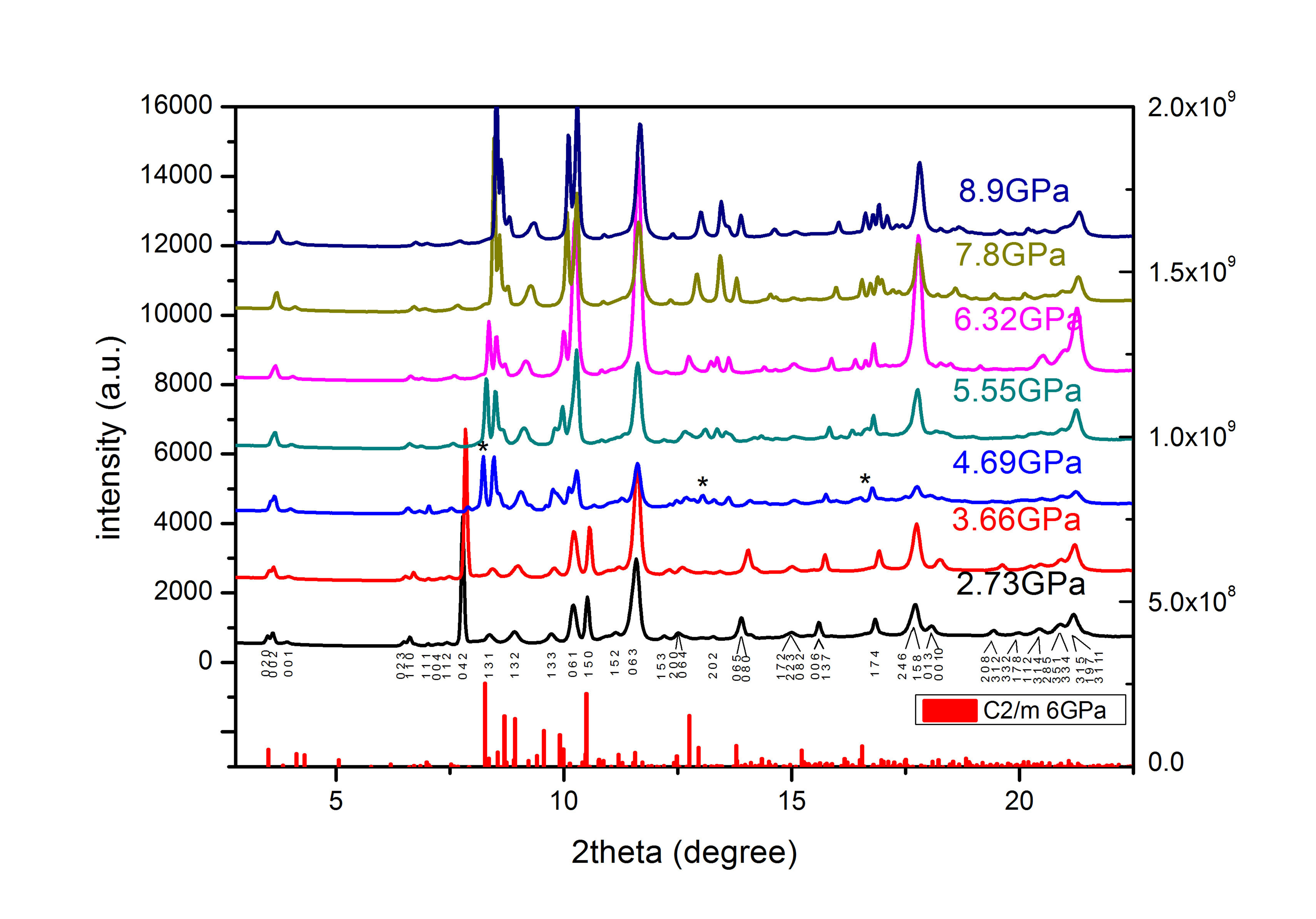


**Figure S3**: The evolution of x-ray diffraction of HfTe_5_ with pressure: New peaks marked with star appeared at 4.69GPa that indicated a phase transition in well consistent with the theoretical calculations.
